# Assessing populations with access to National Cancer Institute-funded sites using local distance-based service areas

**DOI:** 10.1017/cts.2025.10148

**Published:** 2025-09-09

**Authors:** Sharon P. Shriver, Liora Sahar, Vanhvilai L. Douangchai Wills, Devon V. Adams, Mark E. Fleury

**Affiliations:** 1 American Cancer Society Cancer Action Network (ACS CAN), Washington, DC, USA; 2 American Cancer Society (ACS), Atlanta, GA, USA; 3 Guardant Health, Redwood City, CA, USA

**Keywords:** Cancer, access to screening, GIS, rural-urban, clinical trials

## Abstract

**Introduction:**

Travel distance is a key barrier for patients to participate in clinical trials or receive cancer care. The National Cancer Institute (NCI) is a major funder of cancer research infrastructure through grant programs like the NCI Cancer Center (NCICC) and NCI Community Oncology Research Program (NCORP); however, the majority of US sites that care for people with cancer do not directly receive this funding.

**Methods:**

Through geospatial analysis we examined patient distance to NCI-funded sites and evaluated demographic subgroups to identify potential disparities in access to research opportunities. We assessed whether new NCI support to previously unfunded sites could address identified barriers in access.

**Results:**

NCI-funded sites tend to be in urban centers and are less accessible to low-income or rural patients. Nearly 17% of the US population over 35 years old would have to drive over 100 miles to obtain care at an NCI-funded site; only 1.6% would be beyond that distance when non-funded sites are added. For those below poverty level, the proportions are 20.2% and 1.9%, respectively. Several US regions, including the South and Appalachia, have particularly limited access to NCI-funded sites despite high cancer incidence, and much of the West and Great Plains are distant from any cancer facilities.

**Conclusions:**

NCI could address travel distance as a major barrier to research participation by expanding the geographical footprint of its infrastructure funding using existing institutions in areas with identified gaps. Geospatial analysis at the census tract level is recommended and geospatial visualization can help identify strategic areas for interventions.

## Introduction

Cancer is the second leading cause of death in the USA, and a leading cause of death for the US Hispanic population (alongside heart disease) [1]. Travel distance has been reported as a barrier for receiving cancer care [2–4], and differential access to care due to distance may contribute to disparities in treatment and outcomes [5]. A study of patients with cervical and colorectal cancer identified disparities in access to cancer providers among rural, low-income residents, who need to travel significantly longer distances (to see some specialists, over 60 miles), and emphasized the need to increase access to treatment [6]. Patients with non-small cell lung cancer living in areas with low access to specialists were less likely to receive early-stage curative surgery [7] and experienced higher mortality [8].

Research can provide opportunities for patients to access the newest therapies under investigation, and participation in clinical trials is important to advance discovery of treatments for cancer that can improve outcomes. The goal of NCI’s National Cancer Plan states “Every person with cancer or at risk for cancer has an opportunity to participate in research or otherwise contribute to the collective knowledge base, and barriers to their participation are eliminated [9].” Travel distance has been shown to be a barrier to clinical trial participation [10]. Unger et al. found that half of patients in cancer clinical trials traveled more than 13 miles [11]; another study found that 72% of general patients traveled greater than 13 miles for care (not just for clinical trials) [12]. The American Cancer Society Cancer Action Network (ACS CAN) Survivor Views survey, a program designed to engage cancer survivors by participation in a regular survey about important policy issues, found that of those willing to participate in a clinical trial, 23% of respondents would not be willing to travel to another facility that was any farther in distance than their usual clinic, and only 30% would be willing to travel an additional 90 minutes or more than to their usual care site [13–15].

A recent review of disparities in cancer occurrence and outcomes in rural US areas revealed that underrepresented racial and ethnic groups in rural areas were more likely to have higher incidence of cancer, less access to treatment, and higher mortality compared to their White counterparts [16]. A study of accessibility to NCICCs found advantages in accessibility in urban areas and notable clustering of the population under the poverty line in areas with lower accessibility [17]. Including NCICC satellite locations improved access among some racial and ethnic groups (Native American, White, and Asian) as well as rural communities [16]. Increasing access within proximity to place of residence and avoiding long travel may alleviate the disparities in cancer burden among low socioeconomic status (SES) and rural populations.

Meeting the NCI’s National Cancer Plan goal that all patients with cancer have an opportunity to participate in research, without barriers to their participation, requires ensuring that all patients have reasonable access to sites where research is being done. Although research can theoretically take place at any location of care, specialized infrastructure and required research resources are more likely to be available at larger academic centers with dedicated research funding. NCI cancer center grants are awarded to sites already exhibiting a critical mass of relevant research, and funding is meant to build further research infrastructure, administrative management, community outreach, and centralized clinical trial management [18]. Such institutions are necessarily larger in size and typically located in large population centers. The NCI created the NCORP program in 2014, with the explicit intention of bringing research into more community settings. Sites receiving one of these two types of grants are subsequently referred to collectively as “NCI-funded sites.” Reflecting the impact of such infrastructure, cancer patients at NCI-funded sites have been shown to have better long-term outcomes compared to those at other (non-NCI funded) sites [19,20], and clinical trial enrollment is five-fold higher at NCI-designated cancer centers compared to community sites [21] despite similar willingness of patients in those two settings to consent to trial participation [22].

Given the observed differences in research participation between NCI-funded sites and other sites, we sought to understand imputed access (assessed as driving distance) to these two categories of sites. We evaluated this access via geospatial analysis and overlaid demographic and cancer incidence data to understand if NCI-funded locations are well positioned to not only engage the overall cancer population in research, but are also representative of demographic subgroups. The inclusion of non-funded sites allows an assessment of whether new NCI support to previously unfunded sites could address identified gaps in access to existing NCI-funded sites.

## Materials and methods

Geospatial analysis is often used to calculate proximity, identify gaps in services and inform public health policies [2,23–27]. We used Esri’s ArcPro^®^ 3.1 for spatial analysis and maps were evaluated for common color blindness [28].

**Figure 1.**
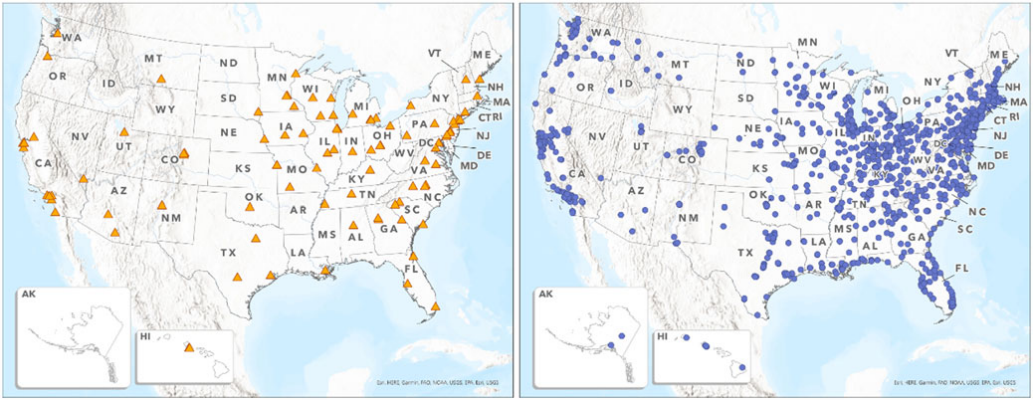
NCICC + NCORP facilities (left). CoC cancer programs (right).

The analysis incorporated multiple datasets including population, mortality and incidence rates, and census tract rural/urban classifications. A list of NCORP locations was downloaded from https://ncorp.cancer.gov/findasite/index.php. Only primary NCORP locations were considered, with the rationale that connected “spoke” sites may not receive fixed or meaningful research funding for research infrastructure, which is reflected in a five-fold difference in enrollment between NCI-designated cancer centers and community sites [21]. NCI also lists over 1,000 NCORP associated sites and including them as “NCI supported” would categorize comprehensive cancer centers receiving millions of dollars annually from NCI together with small community sites that might only receive a few thousand dollars per year based on per-enrollment payments, masking any meaningful comparison of NCI-supported versus non-supported sites. Locations of NCICC were downloaded in June 2022 from the NCI website as a geographic shapefile (https://gis.cancer.gov/ncicatchment/). The American College of Surgeons sponsors a Commission on Cancer (CoC) accreditation program which includes programs offering cancer care across the USA, including NCI-funded sites as well as non-funded sites, which serve approximately 70% of all newly diagnosed cancer patients in the USA [29,30]. The list of CoC-accredited programs was used as a more comprehensive list of care sites (1183 at the time of this analysis) and was downloaded from the organization’s website and geocoded to obtain coordinates (Figure 1). All locations were categorized by rural/urban geographies.

“Rural” and “urban” categories are often used to help identify disparities in access to care and health outcomes [31–37] and various classifications and definitions of “rural” can be applied [34,35,37]. Different patterns of travel are expected between rural and urban environments, and the distance that people are willing to travel to receive a potentially lifesaving treatment varies. Hence, access was defined using multiple increasing distances of 10/20/40/50/100 miles, and greater than 100 miles. Although 100 miles is not a common travel distance, results are presented to illustrate gaps and disparities in services. We utilized road network analysis [23] (preferred over the Euclidean distance method [25,38,39]) to calculate distances and create geographic proximity-based “service areas” around facilities. Distance-based service areas provide a more consistent measure compared with time, since time can be impacted by time of day, weather, road work and conditions, and mode of transportation. Census tracts were designated according to the consolidated rural–urban commuting area (cRUCA) scheme [35]. cRUCA consolidates USDA’s RUCA codes and consists of seven categories, where 1 represents “Urban Core” and 7 represents “Isolated Rural” (Table 1) [40]. Designating rural and urban at the census tract geography enables the identification of disparities and barriers in local communities [34,35,37].

**Table 1.** The distribution of NCICC, NCORP, and CoC programs across rural and urban categories. Total number and percentage of people aged 35+ below poverty with no access within 10, 20, and 40 miles. “Percent” is the percentage of people within the cRUCA.

			Patients aged 35+ below poverty with no access to NCI or CoC within 10 miles		Patients aged 35+ below poverty with no access to NCI or CoC within 20 miles		Patients aged 35+ below poverty with no access to NCI or CoC within 40 miles	
cRUCA	Locations of NCICC + NCORP sites	Locations of CoC sites	Total	Percent	Total	Percent	Total	Percent
1 - Urban core	105 (97.2%)	1003 (84.9%)	7,017,586	57.5%	4,588,770	37.6%	2,580,031	21.2%
2 - Close proximity to urban core	–	23 (1.9%)	1,766,969	94.3%	1,482,584	79.1%	886,025	47.3%
3 - Urban cluster	2 (1.9%)	132 (11.2%)	1,546,936	92.2%	1,465,359	87.4%	1,167,858	69.6%
4 - Close proximity to urban cluster	–	1 (.1%)	40,739	96.3%	37,191	87.9%	31,260	73.9%
5 - Small town	1 (.9%)	19 (1.6%)	821,365	97.1%	780,483	92.3%	618,651	73.1%
6 - Close proximity to small town	–	–	14,883	93.6%	12,982	81.6%	10,089	63.4%
7 - Isolated rural areas	–	4 (.3%)	587,581	97.8%	558,693	93.0%	451,140	75.1%
Total	108	1182	11,796,059		8,926,061		5,745,054	

We estimated the number of people with access to NCICC + NCORP facilities before and after incorporating additional CoC-accredited facilities for all distances and across rural and urban categories for the following population groups:

- People aged 35+
- People aged 35+ living below poverty
- Racial and ethnic groups aged 35+
  - White, Not Hispanic
  - Hispanic
  - Black
  - Not White

While cancer incidence is higher in populations aged 55 and older, we chose age 35 as a cutoff to reflect the growing incidence of early onset cancers [41]. Population estimates for different groups were downloaded from the US Census (2016–2020 American Community Survey 5-year Estimates) [42]. The entire census tract population is considered as either having access or not having access within the distance from the facility. Data were aggregated and reported for service areas showcasing the additional population groups with access at increased distances. We illustrate observed patterns and potential disparities of access to clinical trials nationwide and in rural–urban settings.

Service areas were also overlaid with mortality and incidence rates of all cancers and visual inspection helped identify gaps and areas of higher mortality and incidence rates in regions that do not have access within the service areas.

## Results

### Nationwide analysis

Across all analyzed population groups, the majority (at least 65%) reside within the urban core (cRUCA-1) category and over 80% reside within the urban core or near an urban core (cRUCA-2). We further calculated the number and percentage of people with no access across population groups by cRUCA. Table 1 provides the distribution of NCICC, NCORP, and CoC-accredited facilities across the rural/urban categories, illustrating the larger presence of cancer infrastructure within the more urban categories. Over 97% of NCICC + NCORP facilities are in cRUCA-1. In comparison, 85% of CoC facilities are in cRUCA-1. Only three NCICC + NCORP sites (in New Hampshire, Tennessee, and Wisconsin) are outside of cRUCA-1, compared with 179 CoC sites.

**Figure 2.**
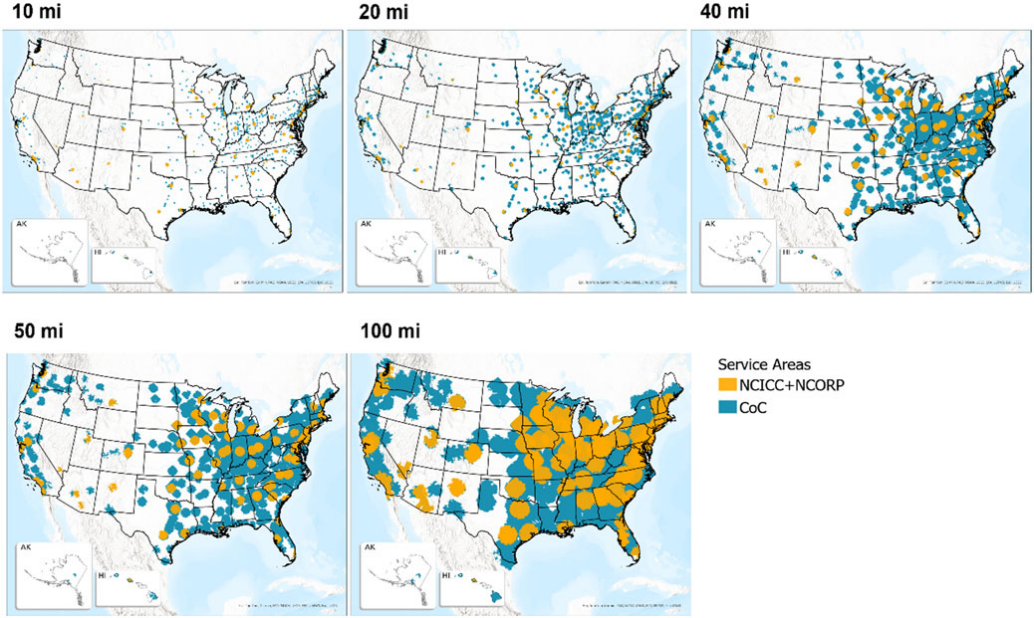
Service areas around NCICC + NCORP (orange) and CoC (blue).

Figure 2 shows service areas around NCICC + NCORP (orange; top layer) and CoC sites (blue; bottom layer) for the various distances. The figure demonstrates the extent of the geographic increase in access to CoC locations across all distances. Some states, i.e., Idaho, Wyoming, and South Dakota, have no or few facilities, hence, low access to potential clinical trials, even within 100 miles. The actual increase in access for each population group within the incremental distances is reflected in Table 2. The table provides the percent of people who do not have access to NCICC + NCORP infrastructure and the percent after adding CoC programs. All census tracts within each distance-based service area were aggregated to calculate the number and percent of people who have and do not have access to facilities within the specific distance. Nationwide, at a 100-mile distance, adding CoC facilities has the potential to decrease the percentage of people with no access to clinical trials across all population groups, from 13%–20% to approximately 2%, based on NCICC + NCORP facilities only. Forty miles is a common distance used for determining access to services [27,43], and at this distance, the number of people without access to NCICC + NCORP sites decreases by 9–14% when CoC facilities are added. Due to the variation in the number of people within each population group, one percentage point can represent from over 170K (adults aged 35+ below poverty) to over 1.7M (adults aged 35+) individuals (1% of each population group is provided in Table 2 as a reference).

**Table 2.** Percent of people within each category who do not have access within the different distances. For each population category and distance, there are two columns representing the percent of people who do not have access to the NCICC + NCORP infrastructure, and the percent who do not have access after adding CoC programs. The percentage is calculated using the total number of people within the population group with no access within the distance-based service areas divided by the total number of people within each population group nationwide.

	Not within 10 miles (%)		Not within 20 miles (%)		Not within 40 miles (%)		Not within 50 miles (%)		Not within 100 miles (%)	
Population group (1% in number of people)	NCI + NCORP	Adding CoC	NCI + NCORP	Adding CoC	NCI + NCORP	Adding CoC	NCI + NCORP	Adding CoC	NCI + NCORP	Adding CoC
**35+** (1% ∼ 1.77M)	79.9	73.2	62.3	53.1	43.6	31.5	37.8	24.7	16.8	1.6
**35+ Below Poverty** (1% ∼173K)	75.9	68.4	62.1	51.7	47.4	33.3	41.9	26.5	20.2	1.9
**35+ Black** (1% ∼202K)	68.0	61.8	49.9	42.2	35.0	24.9	30.8	19.9	13.1	0.4
**35+ Hispanic** (1% ∼247K)	71.6	64.9	51.0	42.1	34.1	22.9	29.8	17.8	16.2	2.3
**35+ White Alone, Not Hisp** (1%∼1.18M)	84.9	77.9	69.0	59.1	49.2	35.9	42.4	28.1	18.2	1.6
**35+ Not White** (1% ∼447K)	68.2	62.7	47.0	40.0	31.0	22.0	27.1	17.4	12.6	1.3

As depicted in Table 2, the inclusion of CoC sites has a greater impact at longer distances, with the difference in the percentage of people aged 35+ with access to potential clinical trial sites ranging from 7% at 10 miles to 15% at 100 miles.

### Access in rural and urban areas

**Figure 3.**
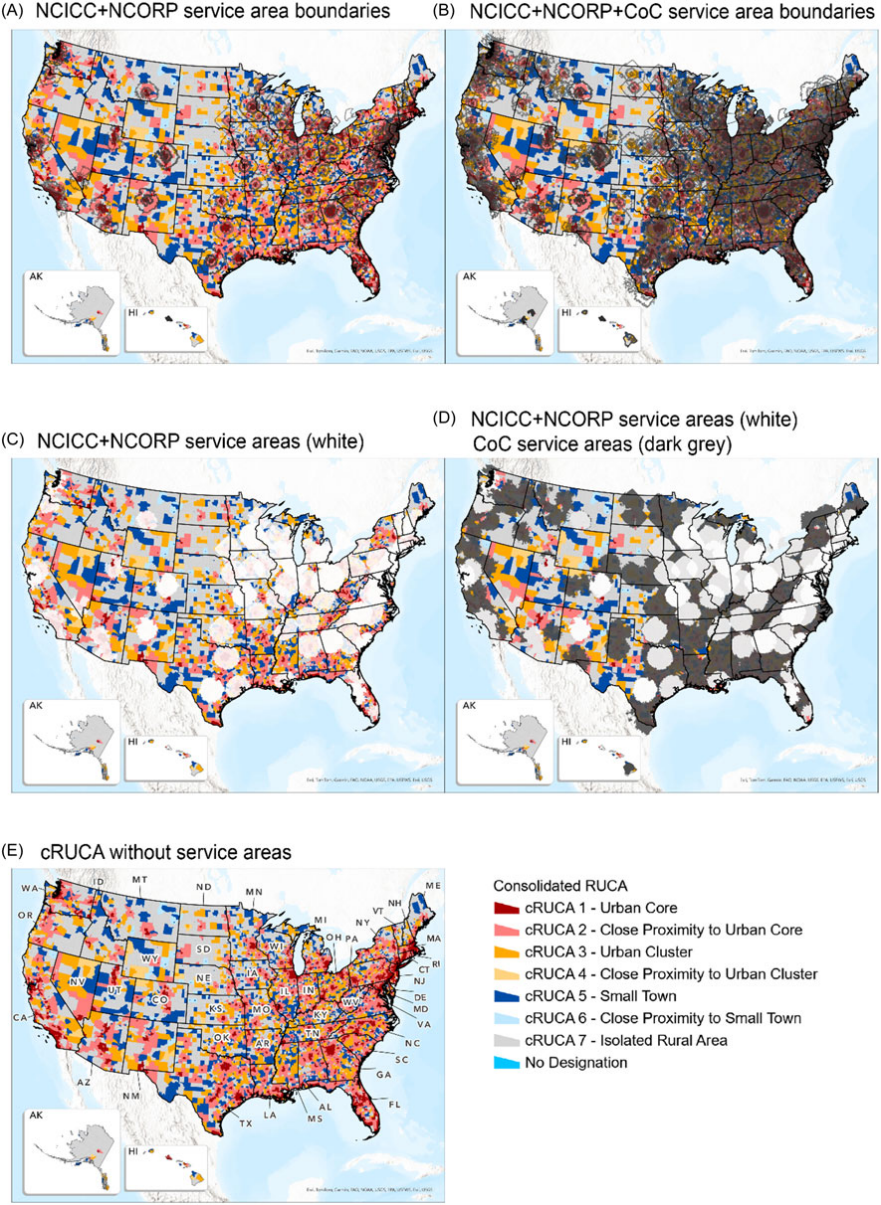
Service areas over cRUCA census tracts map. The cRUCA map (E) serves as a reference showing a rural–urban schema. The left maps show the service areas of the NCICC + NCORP facilities (A and C) and the right maps depict the NCICC + NCORP + CoC facilities (B and D). The top maps (A and B) show the service areas as boundaries and the bottom maps (C and D) show the service areas shaded (white for NCICC + NCORP service areas and dark gray for CoC service areas) for a better illustration of the entire geographic coverage of these service areas. The colors of the geographies that remain visible, unobscured by the overlay of service areas, indicate regions outside the service areas, which are mostly less-urban geographies. It is evident that the NCICC + NCORP service areas are primarily in urban areas and that there is better coverage when CoC facilities are added (right column).

We further examined the geographic access among rural and urban designated census tracts based on the cRUCA schema (Figure 3E). Figure 3 illustrates the service areas for NCICC + NCORP facilities (left panel; white areas at the bottom) and when adding CoC facilities (right panel; dark gray areas at the bottom) both as boundaries (top) and shaded (bottom). The “colored” census tracts visible from under the shaded service areas are the associated cRUCA categories where there is no access. The maps show increased geographic access across all cRUCA categories when adding CoC programs. The maps also depict the concentration of facilities within urban core geographies (covering the red-shaded census tracts) and the increasing access in other categories at longer distances and after adding CoC facilities.

We examined the differential impact of adding CoC programs as a function of cRUCA categories (graphs showing the impact are also provided in the appendix). As expected, a trend of increase in access is evident as distance increases and more people have access. Noteworthy are the higher percentages of people with no access across all cRUCA categories, even at 100 miles within NCICC + NCORP service areas compared with CoC service areas.

Across all geographies and nationwide, there is a clear and consistent trend of more people within the service areas when CoC programs are added. Table 2 depicts the national trend of the percentages of people with no access and shows that approximately 2% of individuals across the population groups do not have access within 100 miles when CoC programs are added. The national trend and cRUCA-1 trend are well aligned, driven mainly by the access rate in urban core areas where most facilities and people reside.

Access increases also in less-urban geographies with the addition of CoC programs that are not in the urban core. Because there are only a few NCICC + NCORP facilities outside of urban core geographies, non-NCI programs drive most of the increase in access in those geographies. There is a notable difference in lack of access between the more urban geographies (cRUCA-1 and -2) and less-urban geographies (cRUCA-3 through -7), particularly within the short-distance service areas of 10 and 20 miles (and 50 miles in some instances) with greater access at 100 miles. cRUCA-2 represents census tracts that are near urban core areas (cRUCA-1). As a result, service areas around the facilities in urban core areas extend to census tracts in close proximity and can explain the greater differences with cRUCA-4 and -6.

At 100 miles, including CoC sites, the percentage of people with no access across cRUCA categories and most population groups is below 10%, aggregated nationwide at about 2% with no access (Table 2). In comparison, for NCICC + NCORP, across all cRUCA categories and population groups, those percentages are above 10% indicating the higher proportion of people with no access, with nationwide aggregations indicating between 13%–20% with no access. Notably, for cRUCA-4 and cRUCA-6, most of the population groups (ranging between 57%–76%) do not have access at 100 miles. Access increases with the addition of CoC programs.

Due to the differences in the size of populations living in the different cRUCA categories, it is important to examine not only the percentage of the population without access, but also the absolute number of people who do not have access. For example, a large percentage (over 90%) of the group aged 35+ below poverty does not have access within the 10-mile service areas (Table 1) in cRUCA-2 through -7, compared with the urban core, cRUCA-1 category (less than 60%). Yet, when reviewing the actual number of people with no access, over 7M people within cRUCA-1 do not have access within 10 miles compared with about 4.8M for all cRUCA-2 through -7 codes combined and less than 2M for each individual category. Table 1 provides additional statistics for the population group across rural and urban geographies, where at 10 and 20 miles, the greater majority within all categories besides cRUCA-1 do not have access, emphasizing the disparity outside of the urban core, with some improvement at 40 miles.

A similar trend is observed across the other population groups based on the distribution of people across the geographies.

### Access and cancer burden

**Figure 4.**
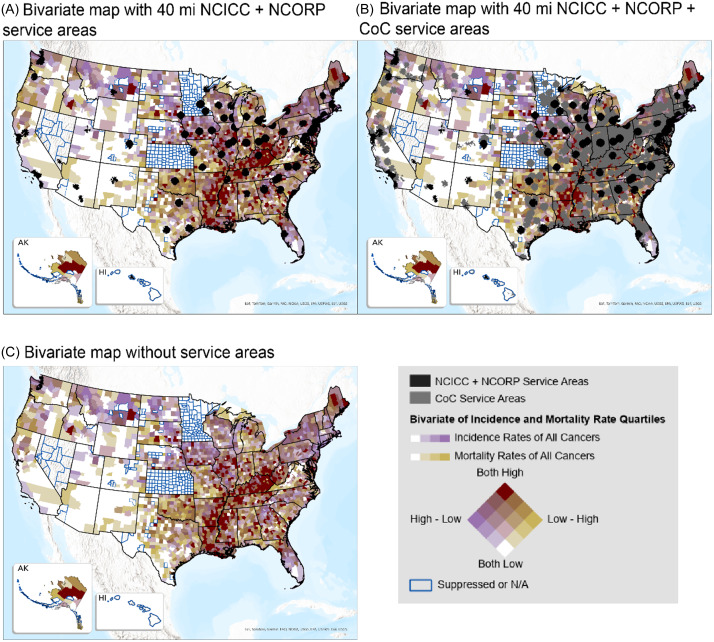
Bivariate map of incidence and mortality rates of all cancers. Incidence and mortality rates of all cancers are shown using quartiles overlaid with polygons depicting the service areas of NCICC + NCORP locations in black and CoC service areas in gray. Counties with suppressed incidence rates are outlined in blue. The top maps show service areas for 40 miles (A and B) and the bottom map (C) shows the rates without the service areas. Geographic areas depicting high mortality and incidence rates are clearly depicted in burgundy in parts of Appalachia and the lower Mississippi Delta. The colors of the geographies that remain visible, unobscured by the black and gray overlay of service areas, indicate regions outside those service areas.

Figure 4 presents bivariate maps, depicting mortality and incidence rates by quartiles of all cancers, overlaid with service area of 40 miles around NCICC + NCORP facilities (black) and CoC (gray). The matrix in the legend shows colors representing areas of high and low mortality rates (yellow shades) and incidence rates (purple shades). Counties with suppressed rates are outlined in blue. Geographies that remain visible, unobscured by the black and gray overlay of service areas, indicate regions outside the 40-mile service areas and their color indicates low/high rates of mortality and/or incidence.

At 40 miles, areas of high mortality and incidence with no access to NCICC + NCORP facilities are depicted in burgundy and found in parts of Appalachia (eastern states from Alabama to Virginia and West Virginia) and parts of the lower Mississippi Delta (Louisiana, Mississippi, and Arkansas). While non-NCI programs provide increased access at 40 miles, there are still pockets of high burden with no access. Other notable pockets are in Maine and states in the southeast including South Carolina, Georgia, and Florida. There are large geographies with no access in the western half of the country, mainly in the central west, such as in Utah, Wyoming, Idaho, Montana, and the Dakotas, that have no or a low number of facilities, with counties of high mortality (burgundy/darker yellow) and/or high incidence (darker purple).

## Discussion

The NCORP program was created in 2014 to bring research into more community settings. These locations serve to augment and complement the care offered at NCICC, which are more likely to be located in urban centers. The NCORP program replaced two other programs, the Community Clinical Oncology Program (CCOP) and the NCI Community Cancer Centers Program (NCCCP). Fourteen of the 46 NCORP sites are minority/underserved designated, where at least 30% of the population served comprises racial/ethnic minorities or rural residents. Despite these efforts to bring research into more communities, our findings indicate that imputed service areas of NCI-funded sites are still skewed to serve people living in urban settings and leave individuals with cancer in rural areas and several geographic pockets in the south and Appalachia with less access. Our results also reinforce earlier findings that populations served by NCI-funded sites are wealthier and less diverse [44].

These findings have implications for representation in research that involves patients, such as tumor biobanking and clinical trials. While non-NCI-funded institutions do perform research, many forms of research require specialized infrastructure and resources. This is especially true for clinical trial access: non-NCI-funded oncology sites exist in locations that are not served by NCI-funded sites, but increasing their potential to accrue patients to clinical trials will require investments such as additional grant programs from NCI. Other studies have found that a much higher proportion of non-metropolitan counties lacked any cancer clinical trials when compared to metropolitan counties [45].

In a recent survey, the top five barriers reported by providers to opening trials included contracting and paperwork burden, lack of staff, lack of relevant patients, lack of financial resources, and lack of infrastructure [46]. Of these five, all but patient availability are tied to resource needs, reflecting how critical financial support is for expansion of research capacity in the community. In 2024, NCI leadership launched an initiative to create recommendations for improving clinical trial access in community and rural settings [47], and among their recommendations were an expansion of the NCORP program and creation of smaller grant program to prepare sites for NCORP status [48]. For such an approach to work, newly funded sites would have to serve populations that currently have limited access to NCI-funded sites. Our findings suggest that additional sites exist that, if augmented through receipt of NCI support, could help address current access issues. Such target areas are visually identified in parts of Appalachia, Louisiana, Florida and other geographies mainly in the eastern half of the country by leveraging geospatial visualization that integrates areas of low or no access with cancer surveillance data. Also noted are areas in the western half of the country and other pockets such as parts of the lower Mississippi Delta that can benefit from other interventions such as newly funded sites. Geospatial analysis should be implemented at the census tract level and aggregated as needed to counties, states, and regions for decision making. Integrating areas of low or no access with variables such as cancer burden and population can help strategically reach specific populations and address needs in communities.

There are some limitations in this study. We only assessed patient proximity to sites and did not factor in site capacity. For example, it would be difficult for lower-volume sites to offer clinical trials for more rare cancers or subtypes, a limitation that could be addressed through a focus on only common cancers. Future analysis that incorporates clinical trial data with measures such as type of cancer, recruitment requirements and capacity would enhance the identification of geographic areas lacking in specific cancer-related services and better address the needs of those communities. NCICC primary locations were used, and this analysis may not have accounted for satellite locations. Additional cancer treatment facilities exist that are not represented in the NCICC, NCORP or CoC institution lists and therefore are not captured in this analysis. Our analysis excluded age ranges that would capture pediatric or many young adult individuals with cancer. Additional financial, cultural, insurance coverage limitations, transportation, and other barriers also play roles in access. For example, some sites may not participate in Medicaid, limiting access for the approximately 20% of patients who utilize Medicaid coverage [49]; a recent study examining Medicaid acceptance at a random sample of CoC facilities found that comprehensive community cancer programs were significantly less likely to provide access to care for patients with Medicaid than NCI-designated cancer programs [50]. This is likely to exacerbate disparities in clinical trial participation and cancer care, given increases in Medicaid coverage of historically disadvantaged populations of patients with cancer, including members of racial and ethnic minority groups, those residing in rural areas, and individuals with lower educational level [49].
